# The long‐term intake of milk fat does not significantly increase the blood lipid burden in normal and high‐fat diet‐fed mice

**DOI:** 10.1002/imt2.256

**Published:** 2024-12-15

**Authors:** Guang‐Xu Ren, Liang He, Yong‐Xin Liu, Yu‐Ke Fei, Xiao‐Fan Liu, Qiu‐Yi Lu, Xin Chen, Zhi‐Da Song, Jia‐Qi Wang

**Affiliations:** ^1^ Institute of Food and Nutrition Development Ministry of Agriculture and Rural Affairs of the People's Republic of China Beijing China; ^2^ Department of Electronic Engineering, and Beijing National Research Center for Information Science and Technology Tsinghua University Beijing China; ^3^ School of Computer Science and Technology, and School of Intelligence Science and Technology Xinjiang University Urumqi China; ^4^ Genome Analysis Laboratory of the Ministry of Agriculture and Rural Affairs, Agricultural Genomics Institute at Shenzhen Chinese Academy of Agricultural Sciences Shenzhen Guangdong China; ^5^ Institute of Animal Sciences Chinese Academy of Agricultural Sciences Beijing China

## Abstract

After 10 weeks of feeding C57BL/6J mice with a normal diet (ND) or a high‐fat diet (HFD), a 7‐week intervention with milk fat and whole milk was conducted to assess their long‐term effects on host blood lipid levels. The results showed that milk fat and whole milk did not significantly elevate low‐density lipoprotein cholesterol (LDL‐C) in either ND‐ or HFD‐fed mice. In ND mice, milk fat and whole milk improved gut microbiota diversity and Amplicon Sequence Variants. Key bacterial genera, such as *Blautia*, *Romboutsia*, and *Prevotellaceae_NK3B31_group*, were identified as bidirectional regulators of LDL‐C and high‐density lipoprotein cholesterol (HDL‐C). Six unique metabolites were also linked to LDL‐C and HDL‐C regulation. Furthermore, an optimized machine learning model accurately predicted LDL‐C (*R*² = 0.96) and HDL‐C (*R*² = 0.89) based on gut microbiota data, with 80% of the top predictive features being gut metabolites influenced by milk fat and whole milk. These findings indicate that the long‐term intake of milk fat does not significantly increase the blood lipid burden, and machine learning algorithms based on gut microbiota and metabolites offer novel insights for early lipid assessment and personalized nutrition strategies.

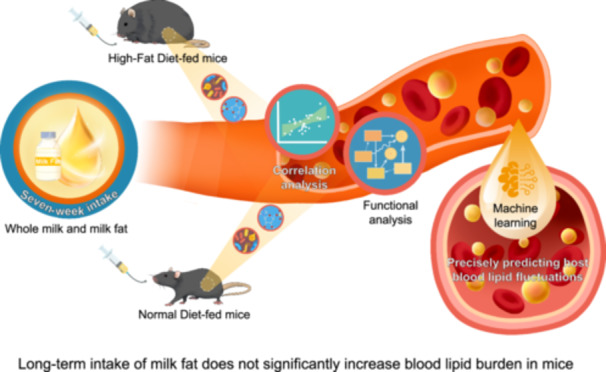

Whole milk is consumed by over 6 billion people around the world, making it the most significant source of food globally [1]. Early observational studies have suggested that diets rich in saturated fatty acids (FA) are positively associated with the increased risk of cardiovascular disease (CVD) [2]. Whole milk, rich in milk fat, is commonly believed to contribute to obesity and CVD, as milk fat is thought to burden the host's blood lipid levels in a way similar to dietary fats. In light of previous epidemiological data suggesting that whole milk consumption increases the risk of coronary heart disease [3], some dietary guidelines recommend reducing the intake of milk fat as a preventive measure against cardiovascular disease. These conventional guidelines have failed to account for the implications of diverse food sources in the diet and the effects that they have by different fats in foods [4]. With more in‐depth research, an increasing number of studies suggested that milk fat contains many beneficial components for human health [5, 6], such as monounsaturated, polyunsaturated, omega‐3, and medium‐chain saturated fatty acids. Furthermore, a large multinational cohort study of individuals enrolled from 21 countries on five continents reported that dairy consumption was associated with a lower risk of mortality and major cardiovascular disease events in a diverse multinational cohort [7]. In addition, a growing body of evidence suggests that increasing the intake of dairy fat does not increase the risk of CVD [8] and may even reduce the risk of central obesity [9]. Therefore, the relationship between long‐term intake of milk fat and the host's blood lipid metabolism remains controversial [10, 11]. The emergence of these contradictory conclusions is primarily due to the complex genetic background of humans, inaccurate dietary monitoring, and differences in factors involved in post‐hoc studies, leading to conflicting conclusions among research teams [12].

It is worth noting that both milk fat and whole milk contain potentially beneficial compounds, many of which might also affect health outcomes. These compounds can reshape the host intestinal microenvironment [13], thereby exerting a certain impact on the secretion network of metabolites throughout the entire gut. The gut microbiota (GM) has been implicated in obesity and metabolism [14], while it has also been shown that the microbiota and metabolites are substantial drivers of circulating lipid levels [15]. Thus, this raises the question of whether long‐term consumption of milk fat can increase the blood lipid burden in the hosts with both normal and obese conditions, and whether the intestinal microenvironment plays a role in this process.

To provide novel insights, we utilized a genetically well‐characterized mouse model with a controlled diet to evaluate the impact of long‐term milk fat and whole milk consumption on blood lipid levels in both normal diet (ND) and high‐fat diet (HFD) groups. Our findings indicate that prolonged consumption of milk fat and whole milk does not significantly increase blood lipid burden and also improves gut microbiota structure. Milk fat and whole milk modulate low‐density lipoprotein cholesterol (LDL‐C) and high‐density lipoprotein cholesterol (HDL‐C) levels bidirectionally by targeting key gut bacteria and metabolites. These alterations in the gut analyzed via an optimized machine learning algorithm, enabled accurate prediction of host blood lipid dynamics.

## RESULTS AND DISCUSSION

### Effects of long‐term milk fat intake on blood lipid profiles in mice fed normal and high‐fat diets

**FIGURE 1:**
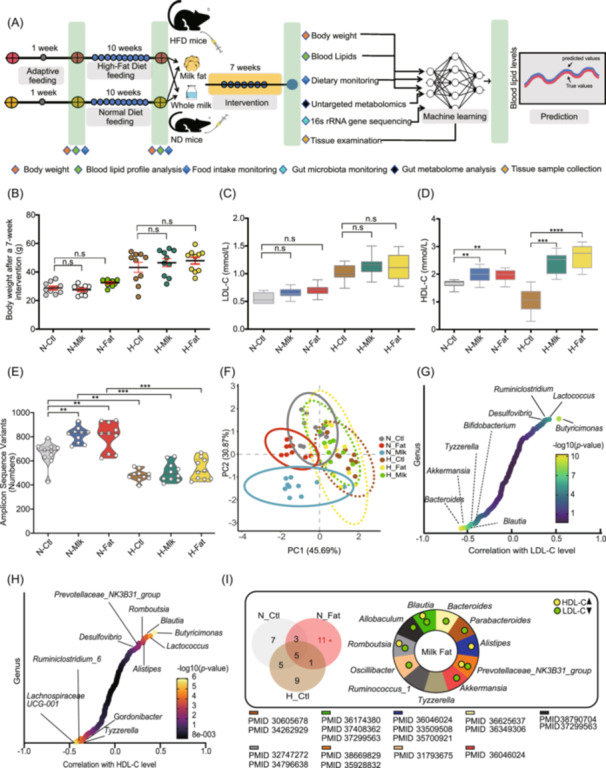
Effects of long‐term intake of milk fat and whole milk on blood lipid levels and gut microbiota in mice fed a normal diet or a high‐fat diet. (A) During a 1‐week acclimation, 8‐week‐old SPF male C57BL/6J mice were fed a standard rodent diet and water *ad libitum*. Subsequently, they were randomly assigned to either a normal diet (ND) or high‐fat diet (HFD) group for 10 weeks, during which blood lipid parameters, including low‐density lipoprotein cholesterol (LDL‐C) and high‐density lipoprotein cholesterol (HDL‐C), were measured. The mice were subsequently divided into six subgroups (*n* = 10) for a 7‐week intervention: ND control (N‐Ctl), ND whole milk (N‐Mlk), ND milk fat (N‐Fat), HFD control (H‐Ctl), HFD whole milk (H‐Mlk) and HFD milk fat (H‐Fat). Additional components, including whey protein (Whp), casein (Cas), and lactoferrin (Ltf), were also included. Details are provided in the supplementary materials. At the sacrifice endpoint, body weight, blood lipid levels, adipose tissue, gut microbiota, and fecal metabolites were analyzed. These data will be utilized to train and validate machine learning algorithms. After a 7‐week intervention with milk fat and whole milk in ND and HFD mice, changes in body weight (B), LDL‐C (C), HDL‐C (D), and gut microbiota ASVs (E) were evaluated. One‐way ANOVA was used for comparisons among more than two groups. *, **, ***, and **** indicate *p* < 0.05, *p* < 0.01, *p* < 0.001, and *p* < 0.0001. Data are presented as mean ± SEM. (F) Principal coordinate analysis illustrates the impact of long‐term milk fat and whole milk intake on gut microbiota composition in ND and HFD mice. Spearman's correlation analysis between the relative abundance of gut microbial genera and LDL‐C (G) and HDL‐C (H), respectively, with results displayed in gradient colors (−log_10_ (*p*‐value)). (I) A Venn diagram illustrates the unique genus‐level functional bacteria associated with blood lipid indicators following milk fat intervention, with functional insights into LDL‐C regulation presented in the pie chart. Research findings are cited using PMID numbers.

To assess the effects of long‐term milk fat and whole milk on blood lipid profiles in normal and high‐fat diet‐fed mice, we induced hyperlipidemia in C57BL/6J mice with a 10‐week high‐fat diet, resulting in increased body weight and hyperlipidemia with elevated LDL‐C and reduced HDL‐C (Figure 1A and Figure S1A–D). During the 7‐week intervention, milk fat, whole milk, and other components did not increase body weight in either ND or HFD mice, despite a significant reduction in weekly food intake in ND mice fed whole milk (Figure 1B and Figure S1E,F). Importantly, LDL‐C levels remained stable in both groups (Figure 1C). Milk fat significantly lowered triglycerides in ND mice (*p* < 0.01), while whey protein reduced triglycerides in both ND (*p* < 0.001) and HFD mice (*p *< 0.05) (Figure S1G). Milk‐related interventions did not cause significant differences in total cholesterol for both ND and HFD mice (Figure S1H). Additionally, both milk fat and whole milk significantly increased HDL‐C levels in ND and HFD mice (Figure 1D), reducing the risk of atherosclerotic cardiovascular disease. Dissection revealed that milk fat and other components did not increase epididymal white or brown adipose tissue weight, and in ND mice, milk fat actually reduced epididymal white adipose tissue (Figure S1I,J).

### Milk fat can bidirectionally regulate peripheral LDL‐C and HDL‐C levels by targeting key gut bacteria

Since gut microbiota regulate host metabolism and are influenced by diet, we investigated how milk fat and whole milk affect the gut microbiota network. As expected, a high‐fat diet significantly reduced amplicon sequence variants (ASVs) and gut diversity (Figure 1E and Figure S2A,B). However, long‐term milk fat and whole milk consumption increased alpha diversity in ND mice, though it had little effect on ASVs, OTUs, and alpha diversity in HFD mice, suggesting a stronger influence of the high‐fat diet on gut structure (Figure 1E and Figure S2A,B). We selected the principal components based on a cumulative contribution rate greater than 70% as the threshold. According to this criterion, PC1 can explain 45.69% of the total variance and PC2 can explain 30.87% of the total variance (Figure 1F). These data indicate that the gut microbiota structure under ND and HFD conditions is significantly different (Figure 1F and Figure S2D). HFD mice showed an increase in *Desulfovibrio* and *Ruminiclostridium*, and a decrease in *Alloprevotella* (Figure S2C). While milk fat and whole milk significantly altered the microbial structure in ND mice (Figure 1F), they did not reverse the HFD‐induced changes in gut microbiota. Correlation analysis revealed that ASVs, OTUs, Shannon, and Observed_species were negatively correlated with LDL‐C, total cholesterol, and triglycerides but positively correlated with HDL‐C (Figure S2E).

A Spearman correlation analysis between 185 gut microbiota genera and lipid indicators highlighted that *Blautia*, *Akkermansia*, *Bacteroides*, and *Tyzzerella* were negatively correlated with LDL‐C, while *Blautia*, *Lactococcus*, and *Desulfovibrio* were positively correlated with HDL‐C (Figure 1G,H). We also analyzed the correlation between strains at the genus level and the levels of triglycerides and total cholesterol (Figure S2F,G). Further analysis identified 11 unique bacteria in the milk fat group, including *Blautia*, *Bacteroides*, and *Akkermansia*, which are significantly linked to blood lipids (Figure 1I). Among these, *Blautia*, *Prevotellaceae_NK3B31_group*, and *Romboutsia* are known to regulate both LDL‐C and HDL‐C, promoting healthy blood lipid levels. These findings suggest that, unlike other dietary fats, long‐term milk fat consumption reshapes the gut microbiota, regulates LDL‐C and HDL‐C bidirectionally, and does not significantly increase the host's blood lipid burden.

### Milk fat influences the intestinal metabolite profile, and the differential metabolites modulate host blood lipid metabolism

**FIGURE 2:**
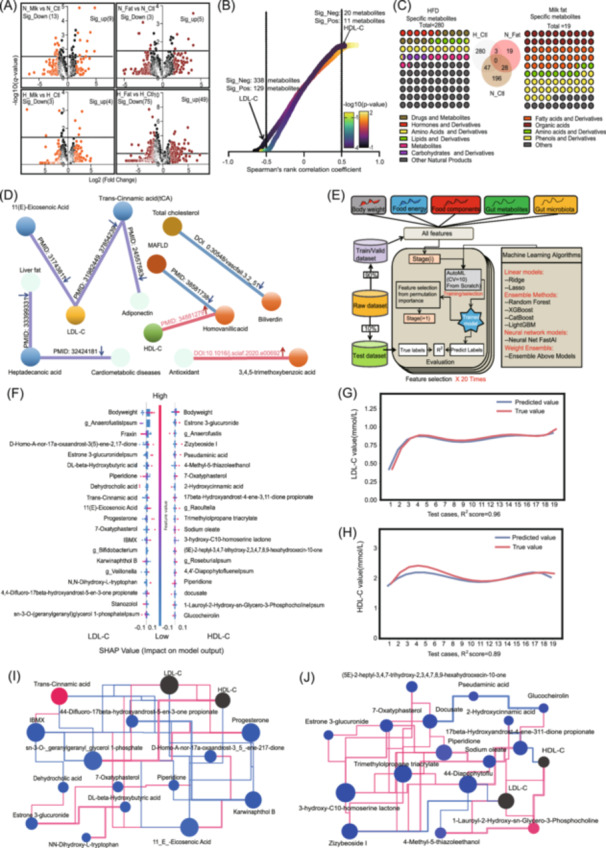
Milk fat and whole milk shape the gut metabolome and enable machine learning‐based prediction of host blood lipid levels using gut microbiota and fecal metabolome data. (A) Volcano plot showing differential gut metabolites in ND and HFD mice after 7 weeks of whole milk and milk fat intervention. Orange and dark red dots represent metabolites with an absolute Log_2_ (Fold Change) >1.5. The dashed line on the *Y*‐axis indicates −log_10_ (*q*‐value) = 1.5. (B) Spearman correlation analysis between gut metabolites and blood lipid levels (LDL‐C and HDL‐C) is shown, with results in gradient colors (−log_10_ (*p‐*value)). The *X*‐axis represents Spearman's rank correlation coefficient, and the *Y*‐axis ranks 3237 metabolites by correlation strength. For HDL‐C, 20 metabolites are significantly negatively correlated (Sig_Neg), and 11 metabolites are significantly positively correlated (Sig_Pos). For LDL‐C, 338 metabolites are significantly negatively correlated (Sig_Neg), and 129 are significantly positively correlated (Sig_Pos). (C) Classification and proportion of unique metabolites induced by milk fat and high‐fat diets. A total of 280 unique metabolites were induced by the HFD, and 19 unique metabolites were induced by milk fat. Metabolite categories are displayed in different colors. The Venn diagram highlights the shared and unique metabolites between the HFD group (H_Ctl) and the milk fat group (N_Fat). (D) Key differential metabolites uniquely induced by milk fat have been previously reported to be associated with lipid metabolism and their functional relationships. (E) Diagram of machine learning training using gut metabolites and microbial structure to predict blood lipid levels. (F) Shapley Additive Explanation (SHAP) analysis identifying 20 key factors influencing the accuracy of LDL‐C and HDL‐C predictions. (G, H) Machine learning predictions of blood lipid levels based on gut microbiota and fecal metabolome data, with blue lines representing predicted values and red lines showing actual values. The Taxi network diagram primarily presents the correlation network between the top contributing metabolites for predicting LDL‐C (I) and HDL‐C (J) levels, as well as their correlation with blood lipid indicators (LDL‐C and HDL‐C, black dots in the network diagram). Red lines represent positive correlations, blue lines represent negative correlations, and the thickness of the lines indicates the degree of correlation. Red dots represent high abundance values, blue dots indicate lower abundance values, and gray dots signify the absence of abundance values.

Given that the host's gut ecosystem primarily regulates host health through metabolites, a nontargeted metabolomics analysis was then conducted to elucidate the effects of metabolites on host blood lipid levels. In our analysis of 1177 metabolites, whole milk in ND mice upregulated 9 metabolites and downregulated 13 metabolites, while milk fat upregulated 5 metabolites and downregulated 3 metabolites (Figure 2A, Tables S1–4). In HFD mice, whole milk affected 7 metabolites, while milk fat had a stronger impact, upregulating 49 metabolites and downregulating 75 metabolites. These results suggest that milk fat more effectively reshapes the gut metabolome in HFD mice than in ND mice. To clarify the relationship between gut metabolites and LDL‐C and HDL‐C levels, we performed a correlation analysis using an absolute correlation coefficient greater than 0.5 as the significance threshold (Figure 2B). Notably, 338 metabolites were negatively correlated with LDL‐C, while 129 metabolites showed a positive correlation. For HDL‐C, 20 metabolites had a negative correlation, and 11 metabolites had a positive correlation. Additionally, beta‐Estradiol and savinin showed significant positive correlations with total cholesterol and Chao1, respectively, while rosmarinine and l‐Cysteine‐S‐sulfate had significant negative correlations (Figure S3A,B). We also found that 3‐methyladenine has a significant negative correlation with triglycerides (Figure S3C). Further analysis revealed that the HFD resulted in 280 specific differential metabolites, whereas milk fat induced 19 specific differential metabolites (Figure 2C). These 19 differential metabolites induced by milk fat mainly consist of fatty acids, phenols, and their derivatives. Functional analysis of differential metabolites in each group revealed that metabolites from whole milk and milk fat interventions, such as cis‐5,8,11,14,17‐Eicosapentaenoic acid, beta‐Estradiol, adrenosterone, tyrosine, progesterone, and others, are involved in taurine and hypotaurine metabolism, steroid hormone and biosynthesis, amino acid metabolism, linoleic acid metabolism, and the biosynthesis of unsaturated fatty acids (Figure S3D). Among the 19 differential metabolites, 11(E)‐Eicosenoic acid and Trans‐Cinnamic acid (tCA) reduce LDL‐C, with tCA also lowering adiponectin. Homovanillic acid increases HDL‐C and is linked to a lower risk of metabolic‐associated fatty liver disease. Biliverdin reduces total cholesterol, while Heptadecanoic acid is negatively associated with cardiometabolic disease and liver fat. 3,4,5‐Trimethoxybenzoic acid boosts antioxidant capacity (Figure 2D).

### Predicting blood lipid changes using an optimized machine learning algorithm with reshaped gut microbiota and metabolites

Our next objective was to uncover factors driving blood lipid changes and their relationships with whole milk and milk fat intake. We applied an optimized machine learning algorithm to predict blood lipid levels (Figure 2E), finding that body weight significantly influences LDL‐C and HDL‐C predictions. Additionally, Trans‐Cinnamic acid, 11(E)‐Eicosenoic acid, and Fraxin are key for predicting LDL‐C, while 4‐Methyl‐5‐thiazoleethanol, Anaerofustis, and Zizybeoside strongly impact HDL‐C predictions (Figure 2F). Encouragingly, our algorithm, using gut data, accurately predicted LDL‐C (*R*² = 0.96), HDL‐C (*R*² = 0.89), triglycerides (*R* ^2^ = 0.79) and total cholesterol (*R*² = 0.96) (Figure 2G,H and Figure S4C,D). A ternary plot showed that whole milk and milk fat significantly influenced key factors for HDL‐C and LDL‐C predictions (Figure S4A,B). These findings suggest a complex regulatory network among factors used for accurate LDL‐C and HDL‐C prediction, showing that milk fat's impact on blood lipids is broad, with gut factors interrelated and collectively shaping blood lipid levels (Figure 2I,J). Individual core factors and their network collectively influence HDL‐C and LDL‐C outcomes.

Notably, although there were no significant differences in body weight and LDL‐C levels (*p* > 0.05) between ND group and HFD mice during the 7‐week intervention, the mean values in the N‐Mlk, N‐Fat, H‐Mlk, and H‐Fat groups showed a slight increase compared to the control group (Figure 1B,C). This may be because milk fat is composed of various fatty acids, including saturated fats, conjugated linoleic acid (CLA), and trans‐vaccenic acid, each of which has different effects on fat accumulation and LDL‐C [16]. For instance, CLA may reduce obesity, while trans‐vaccenic acid lowers LDL‐C and triglycerides. Our study suggests that milk fat and whole milk may improve a complex regulatory network rather than a single microbe strain or metabolite. This explains why, even after 7 weeks (approximately equivalent to 5‐6 years in humans), no significant differences in body weight or LDL‐C were observed. It is important to note that this study utilized ND and HFD mouse models. However, mice possess two subtypes of apolipoprotein, APOB48 and APOB100, which make them naturally resistant to atherosclerosis [17]. Future studies investigating the effects of milk fat on atherosclerosis should consider using LDLR^−/−^, APOE^−/−^ mouse models, or hamster models, which better mimic human lipid metabolism. Future studies should also include detailed analyses of liver and adipose tissue morphology to provide deeper insights into the underlying mechanisms.

## CONCLUSION

Our research shows that long‐term consumption of whole milk and milk fat does not significantly increase the body weight and blood lipid burden in normal and high‐fat diet‐fed mice. Furthermore, milk fat helps improve gut microbiota diversity and increases the abundance of key bacteria and metabolites involved in lipid regulation. Using an optimized machine learning algorithm, we mapped a unique gut–metabolite network that accurately predicts changes in blood lipid indicators through the host's gut microbiota and metabolite profiles (Figure S5). These findings enhance our understanding of the long‐term effects of milk fat on blood lipid health and provide data support for global nutrition policy development.

## METHODS

Detailed procedures for the research model, experimental design, diet and supplements, blood lipid measurements, microbiome sequencing (Table S5), untargeted metabolomics, machine learning algorithm, and statistical analysis and graphing can be found in the Supplementary Information.

## AUTHOR CONTRIBUTIONS

**Guang‐Xu Ren**: Conceptualization; software; data curation; writing—review & editing; writing—original draft; funding acquisition. **Liang He**: Software; data curation; validation; writing—original draft. **Yong‐Xin Liu**: Software; data curation; visualization; writing—review & editing. **Yu‐Ke Fei**: Data curation; resources; writing—original draft. **Xiao‐Fan Liu**: Software; data curation; visualization; investigation. **Qiu‐Yi Lu**: Software; data curation. **Xin Chen**: Methodology; software; data curation. **Zhi‐Da Song**: Software; data curation. **Jia‐Qi Wang**: Supervision; resources; writing—review & editing; funding acquisition.

## CONFLICT OF INTEREST STATEMENT

The authors declare no conflicts of interest.

## ETHICS STATEMENT

All experimental procedures involving animals were performed in accordance with protocols approved by the Institutional Animal Care and Usage Committees (IACUC) of the Institute of Food and Nutrition Development, MARA (YYSLLSC2020005).

## Supporting information


**Figure S1.** Effects of whole milk components on body weight and blood lipid indicators in ND and HFD mice.
**Figure S2.** Effects of whole milk components on gut microbiota in ND and HFD mice and their relationship with blood lipid levels.
**Figure S3.** Effects of whole milk components on metabolites in ND and HFD mice and their relationship with Lipid metabolism.
**Figure S4.** Prediction of blood lipids based on host gut metabolites.
**Figure S5.** Overall summary diagram.

**Table S1**. Differential changes and q‐value of the metabolites between group H_Fat and group H_Ctl in Figure 2A.
**Table S2**. Differential changes and q‐value of the metabolites between group H_Mlk and group H_Ctl in Figure 2A.
**Table S3**. Differential changes and q‐value of the metabolites between group N_Fat and group N_Ctl in Figure 2A.
**Table S4**. Differential changes and q‐value of the metabolites between group N_Mlk and group N_Ctl in Figure 2A.
**Table S5**. Sequencing primers for the V3‐V4 region of the 16S rRNA gene.

## Data Availability

The sequence data have been deposited in the NCBI Sequence Read Archive under BioProject (PRJNA1108019, https://www.ebi.ac.uk/ena/browser/view/PRJNA1108019). The data and scripts used are saved in GitHub https://github.com/Dr-Rgx/imeta-Milk-fat-and-Blood-Lipid. Supplementary materials (methods, figures, tables, graphical abstract, slides, videos, Chinese translated version, and update materials) may be found in the online DOI or iMeta Science http://www.imeta.science/.
