# Supplementary material for: The long‐term intake of milk fat does not significantly increase the blood lipid burden in normal and high‐fat diet‐fed mice
Source: Imeta. 2024 Dec 15;3(6):e256. doi: 10.1002/imt2.256 (PMC11683457; doi:10.1002/imt2.256)
Supplement: Supplementary file 1 — Figure S1. Effects of whole milk components on body weight and blood lipid indicators in ND and HFD mice. Figure S2. Effects of whole milk components on gut microbiota in ND and HFD mice and their relationship with blood lipid levels. Figure S3. Effects of whole milk components on metabolites in ND and HFD mice and their relationship with Lipid metabolism. Figure S4. Prediction of blood lipids based on host gut metabolites. Figure S5. Overall summary diagram. [file IMT2-3-e256-s002.docx]

# Supporting information to

## Title

# The long-term intake of milk fat does not significantly increase the blood lipid burden in normal and high-fat diet-fed mice

**Running title**

Milk fat does not significantly increase mouse blood lipid burden

Guang-Xu Ren^1^ ^#^， Liang He ^2,3^ ^#^ ，Yong-Xin Liu ^4^ ^#^， Yu-Ke Fei^1^ ，Xiao-Fan Liu^1^ ，Qiu-Yi Lu^3^ ，Xin Chen^3^ ，Zhi-Da Song^3^，Jia-Qi Wang ^1,5^*

^1^Institute of Food and Nutrition Development, Ministry of Agriculture and Rural Affairs of the People’s Republic of China, Beijing, 100081, China

^2^Department of Electronic Engineering, and Beijing National Research Center for Information Science and Technology, Tsinghua University, Beijing, 100084, China

^3^School of Computer Science and Technology, and School of Intelligence Science and Technology, Xinjiang University, Urumqi, 830017, China

^4^Genome Analysis Laboratory of the Ministry of Agriculture and Rural Affairs, Agricultural Genomics Institute at Shenzhen, Chinese Academy of Agricultural Sciences, Shenzhen, Guangdong, 518120, China

^5^Institute of animal sciences, Chinese Academy of Agricultural Sciences, Beijing, 100193, China

# These authors contributed equally: Guang-Xu Ren, Liang He and Yong-Xin Liu.

*Correspondence: wangjiaqi@caas.cn (Jia-Qi Wang)

**Methods**

**Research model**

The 8-week-old specific pathogen-free (SPF) male C57BL/6J mice from Beijing Vital River Laboratory Animal Technology Co., Ltd were used in this study. After arrival, mice were housed at the Beijing Institute of Technology under a controlled 12h:12h light: dark cycle, with regulated humidity and temperature. All experimental procedures involving animals were performed in accordance with protocols approved by the Institutional Animal Care and Usage Committees (IACUC) of the Institute of Food and Nutrition Development, Ministry of Agriculture and Rural Affairs of the People's Republic of China (MARA). The approval number for these procedures is YYSLLSC2020005.

**Experimental Design**

During one week of adaptive feeding, the mice were fed a standard rodent maintenance diet and water *ad libitum*. After adaptive feeding and baseline data collection, mice were randomly assigned to two groups, the normal diet group (ND) and the high-fat diet group (HFD), and were continuously fed for 10 weeks while monitoring blood lipid indicators. After completing the high-fat model, the mice were subsequently divided into six subgroups (*n*=10) for a 7-week intervention: ND control (N-Ctl), ND whole milk (N-Mlk), ND milk fat (N-Fat), HFD control (H-Ctl), HFD whole milk (H-Mlk) and HFD milk fat (H-Fat). Additional components, including whey protein (N-Whp and H-Whp), casein (N-Cas and H-Cas), and lactoferrin (N-Ltf and H-Ltf) were also included. At the sacrifice endpoint, body weight, blood lipid levels, adipose tissue, gut microbiota, and fecal metabolites were analyzed (Figure1 A).

**Normal diet, high-fat diet and milk-related components**

The normal diet (ND) used standard SPF-grade maintenance feed (MCF, GB14924.3-2010, CNS) sourced from Beijing Keao Xieli Feed Co., Ltd., while the high-fat diet (HFD) used high-fat feed (HFC, D12492) from Research Diets Co., Ltd. The HFC (5.24 kcal/g) was 60% fat, 20% carbohydrates, 20% protein, contrasting with the MCF at 11.4% fat, 62.8% carbohydrates, 25.8% protein. Mice were initially fed MCF ad libitum for adaptation. In the subsequent ten weeks, ND and HFD groups received unrestricted MCF and HFC, respectively. For the seven-week intervention, whole milk subgroup mice were given 15 mL/day of Beijing Sanyuan^R^ pasteurized milk (3.7g fat/100g, 3.1g protein/100g, 4.7g carb/100g, 270kj energy/100g). The milk fat subgroup received 0.5 ml/day of Sanyuan^R^ fat (40g fat/100g, 1g protein/100g, 4g carb/100g, 1565KJ energy/100g) via intragastric gavage. Whey protein (Yuanye Biotech^R^, > 80% pure) was administered at 54 mg/mL to the whey subgroup, lactoferrin ( > 95% pure) at 33 mg/mL to the lactoferrin subgroup, and casein ( > 90% pure) at 48 mg/mL to the casein subgroup, all via gavage.

**Blood lipid measurements**

After an 8-hour fasting period, venous blood samples were collected from mice for biochemical analysis of blood lipids. After peripheral blood was separated and left to stand, serum was obtained through centrifugation. The automated biochemical analyzer (Olympus AU2700; Olympus, Hamburg, Germany) was used to analyze and measure the levels of low-density lipoprotein cholesterol (LDL-C), high-density lipoprotein cholesterol (HDL-C), triglycerides (TG), and total cholesterol (TC) in the serum.

**16s rRNA microbiome sequencing**

After intervention, fecal DNA was extracted and sequenced for the V3-V4 region of the 16S rRNA gene using the MiSeq platform (Illumina), following the methods previously published [1]. DNA was extracted from thawed stool samples using an E.Z.N.A. Stool DNA Kit (Omega Bio-tek, Norcross, GA, US) according to the manufacturer’s instructions. The quality of the DNA was evaluated by 2% agarose gel electrophoresis and spectrophotometry. The V3-V4 region of the 16S rRNA gene was amplified using FastPfu DNA Polymerase (TransStartTM, TransGen Biotech).The PCR cycling conditions were as follows: 95°C for 5 min; followed by 28 cycles of 45 s at 95°C, 50 s at 55°C, and 45 s at 72°C; and a final extension at 72°C for 10 min. Purified amplicons were pooled in equimolar amounts and subjected to paired-end sequencing (2×300) using the Illumina MiSeq platform according to standard protocols. The sequence data were compiled and filtered for quality. The unique sequence set was classified into operational taxonomic units (OTUs) with a threshold of 97% identity using UCLUST. Chimeric sequences were identified and removed using Usearch (version 8.0.1623). The taxonomy of each 16S rRNA gene sequence was analyzed with UCLUST by comparison to the Silva 119 16S rRNA database with a confidence threshold of 90%. Taxonomic assignments for each OTU were achieved by similarity using the GLSEARCH program. Alpha diversity values were calculated using Faith’s phylogenetic diversity.

**Metabolome analysis**

Fecal metabolites were resolved utilizing an ultra-high-performance liquid chromatography (UPLC) setup, model 1290 Infinity LC from Agilent Technologies, Santa Clara, CA, United States, interfaced with a quadrupole time-of-flight mass spectrometer, TripleTOF 6600, from AB Sciex, Framingham, MA, United States. Fecal sample analyses were conducted on a 2.1 mm × 100 mm ACQUIY UPLC BEH column with 1.7 μm particles (Waters, Milford, MA, United States). The mobile phase, compatible with both positive and negative electrospray ionization modes, was composed of 25 mM ammonium acetate and 25 mM ammonium hydroxide in water and acetonitrile, respectively. The solvent gradient profile started at 85% acetonitrile for the first minute, decreased linearly to 65% over the next 11 minutes, further reduced to 40% in 0.1 minute, held for 4 minutes, and then ramped back up to 85% in another 0.1 minute, followed by a 5-minute re-equilibration phase. The electrospray conditions were optimized with ion source Gas1 and Gas2 at 60, curtain gas at 30, source temperature at 600°C, and ion spray voltage floating at ± 5500 V. The mass spectrometer was configured to capture m/z signals ranging from 60 to 1000 Da with a time-of-flight MS scan accumulation time of 0.20 seconds per spectrum. In auto-MS/MS mode, the m/z range was set from 25 to 1000 Da, with a production scan accumulation time of 0.05 seconds per spectrum. Information-dependent acquisition in high-sensitivity mode was employed for the production scan, with a fixed collision energy of 35 ± 15 eV and a declustering potential of ± 60 V.

Pooled/intra-study quality control samples are created by pooling aliquots of all biological samples in the study. Metabolite identification was achieved through high-precision mass spectrometry (mass error < 25 ppm) and MS/MS data matching against a reference database. For the ion features extracted, only variables with over 50% nonzero measurements in at least one group were considered. Multivariate statistical analysis was performed using the MetaboAnalyst 1 online platform. After Pareto scaling, principal component analysis (PCA) and orthogonal partial least squares discriminant analysis (OPLS-DA) were executed. Model robustness was assessed through leave-one-out cross-validation and response permutation testing. Metabolites with significant differences between groups were identified by combining a statistically significant threshold of variable influence on projection (VIP) values from the OPLS-DA model with a two-tailed Student’s t-test (*p*-value) on the raw data. Significance for metabolites was determined with VIP values > 1.0, *p*-values < 0.05, and a VIP threshold of less than 0.05.

**Machine learning algorithm**

The data was divided into training and testing sets in a 9:1 ratio, and the model was developed over 20 iterations, involving model and feature selection processes. An autoML framework selected a model from various algorithms and extracted features, employing the permutation importance method to identify the most impactful features on predictions. These features were used to refine the model in subsequent iterations, with its performance monitored through the R^2^ score on the test set. The final model, which achieved the highest R^2^ score, illustrates the machine learning workflow used to predict blood lipids as shown in Figure 2 E. Detailed materials and methods are described below:

**Overall Process:** We split the original data into training and testing datasets in a 9:1 ratio. The model-building process was completed over 20 iterations, each consisting of two parts: model selection and feature selection. These parts worked together to identify features in the data with high biological interpretability. First, we extracted all features from the data and obtained trained models from multiple machine learning algorithms using an AutoML framework. We then used the permutation importance method to select the features with the greatest impact on prediction outcomes and trained the model for the next iteration using those features. The performance of the trained model was tested on the testing dataset, and its R^2^ score was recorded. Through 20 iterations, we obtained the model with the highest R^2^ score. The machine learning workflow for predicting blood lipids is shown in Figure 2 E.

**Model Selection:** We explored multiple models (linear/non-linear, parametric/non-parametric, single/ensemble learning, etc.) to determine the most suitable model for predicting blood lipids. The selection process in each iteration was based on the Stacking CV method in AutoGluon. We used 10-fold cross-validation data to construct meta-features, which were combined with the original features to train the meta-model. Permutation importance was calculated to obtain feature ranking, which was then used for the next iteration of meta-model training.

**Feature Selection:** In addition to exploring various models, we focused on feature selection for our data. Considering the impact of the curse of dimensionality caused by an excessive number of features, the increase in model interpretability by removing irrelevant features, and reduced training and inference times, we conducted permutation importance feature ranking in each iteration to select important features for training the next model. This process helped to enhance the performance of our ensemble model.

**Attribution Analysis:** We explained our ensemble model to identify more specific factors affecting blood lipids. Specifically, we used the Shapley Additive Explanation (SHAP) method for attribution analysis. Compared to earlier methods, the advantage of SHAP values is that they reflect the influence of each feature on each sample, including positive or negative effects. For each predicted sample, the model generates a predicted value, and the SHAP value represents the assigned value for each feature in that sample, referred to as the contribution score. If the contribution score is greater than zero, it indicates a positive effect on the model’s prediction, while a score less than zero indicates a negative effect. During the experiment, we used the KernelExplainer from the SHAP model interpretation package in Python to explain the model predictions as the contribution of each feature to the prediction.

**Statistical Analysis and Graphing**

Statistical analyses were performed in GraphPad Prism 8.2.1 or R software. Statistical details for all tests performed are included in the figure legends. n indicates the number of independent replicas. Oneway ANOVA was used to calculate significance for α-diversity-related analyses. Statistical differences across multiple samples were determined by Kruskal-Wallis test. Test for association across all groups of samples was performed through correlation analysis. This was based on Spearman’s rank correlation test by the R function of cor.test. ome bioinformatics data presentation is obtained using the Wekemo Bioincloud platform[2]. Not significant *p* > 0.05; * *p* < 0.05; ** *p* < 0.01; *** *p* < 0.001, **** *p* < 0.0001.


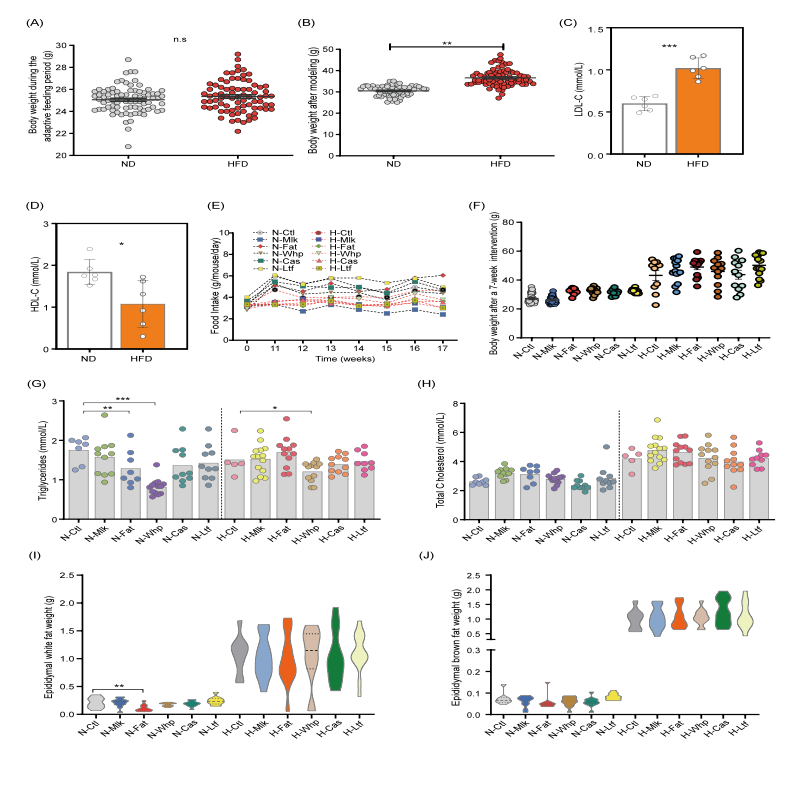


**Figure S1.** Effects of whole milk components on body weight and blood lipid indicators in ND and HFD mice.

(A) Baseline body weights of mice to be included in the ND and HFD groups after the completion of the acclimatization feeding period. (B) Body weight conditions of mice at the end of 10 consecutive weeks of ND and HFD feeding. (C-D) Six mice from each group were randomly selected to measure the levels of low-density lipoprotein cholesterol (LDL-C) and high-density lipoprotein cholesterol (HDL-C) in peripheral blood. (E) Dynamic changes in the weekly spontaneous food intake of mice in each group during the modeling period and the 7-week intervention with whole milk, milk fat, and other main components. After seven weeks of intervention with whole milk, milk fat and other main components of milk in both ND and HFD mice, body weight (F), Triglycerides (G), Total cholesterol (H), epididymal white adipose tissue weight (I), and epididymal brown adipose tissue weight (J) were measured. *, **, ***, and **** indicate *p* < 0.05, *p* < 0.01, *p* < 0.001, and *p* < 0.0001. Data are presented as mean ± SEM.

**
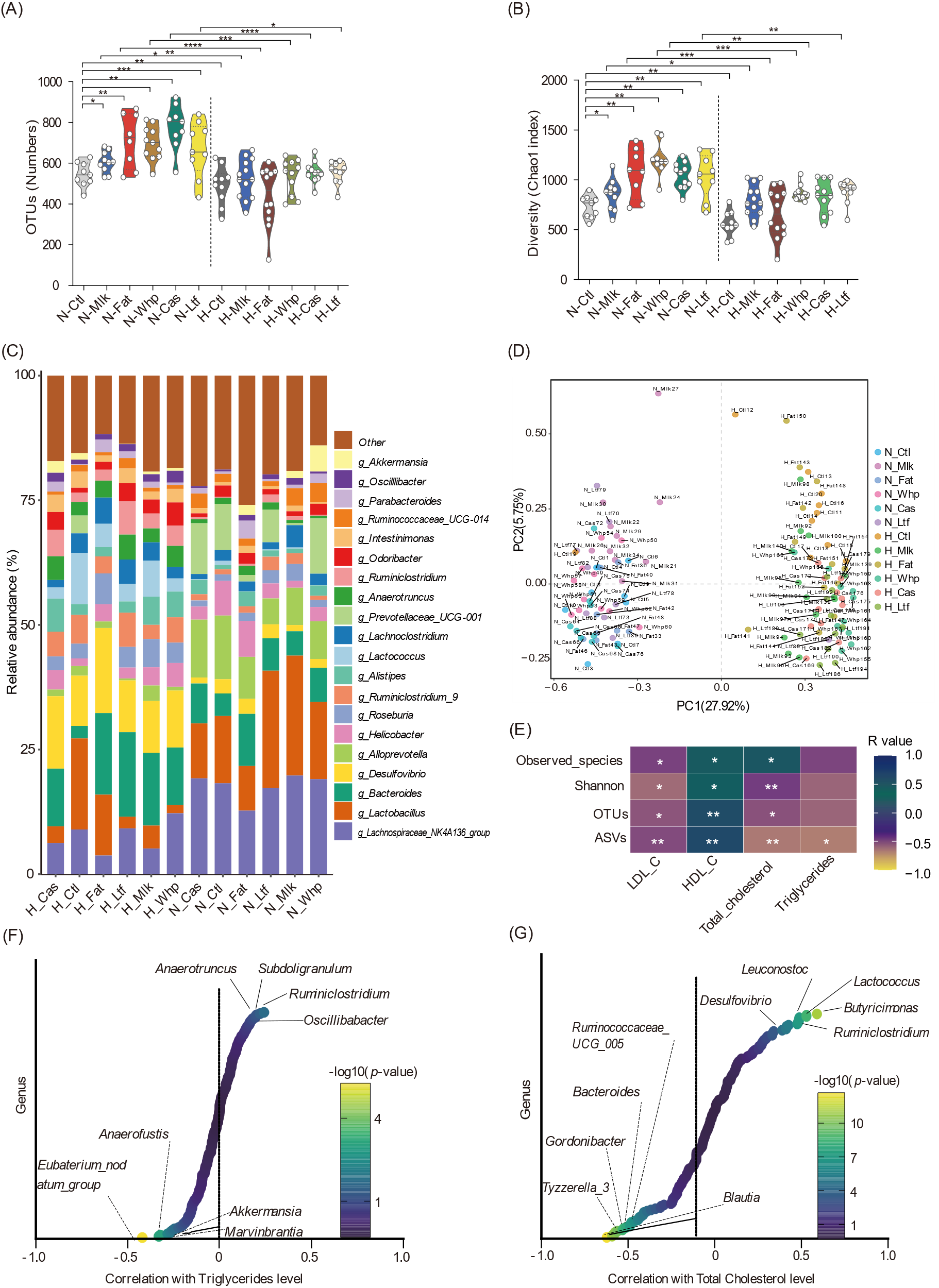
**

**Figure S2.** Effects of whole milk components on gut microbiota in ND and HFD mice and their relationship with blood lipid levels**.**

After seven weeks of intervention with milk fat, whole milk, whey protein, casein, and lactoferrin in both ND and HFD mice, the host gut (A) Operational Taxonomic Units (OTUs) and (B) Chao1 were assessed. (C) The bar plot illustrates the relative abundance of predominant genus-level phylotypes in the gut of ND and HFD mice after a seven-week intervention with whey protein, casein, lactoferrin, whole milk and milk fat. (D) Principal coordinate analysis (PCA) primarily presents the impact of long-term intake of Whey Protein, Casein, and Lactoferrin on the gut microbiota structure of ND and HFD mice. (E) The heatmap displays the correlation coefficients R values of Observed_species, Shannon, OTUs, and ASVs with LDL-C, HDL-C, Total cholesterol, and Triglycerides. The correlation between the relative abundance of gut microbial taxa at the genus level and blood lipid parameter levels including Triglycerides level (F) and Total Cholesterol (G) was analyzed using Spearman’s test. *, **, ***, and **** indicate *p* < 0.05, *p* < 0.01, *p* < 0.001, and *p* < 0.0001. Data are presented as mean ± SEM.


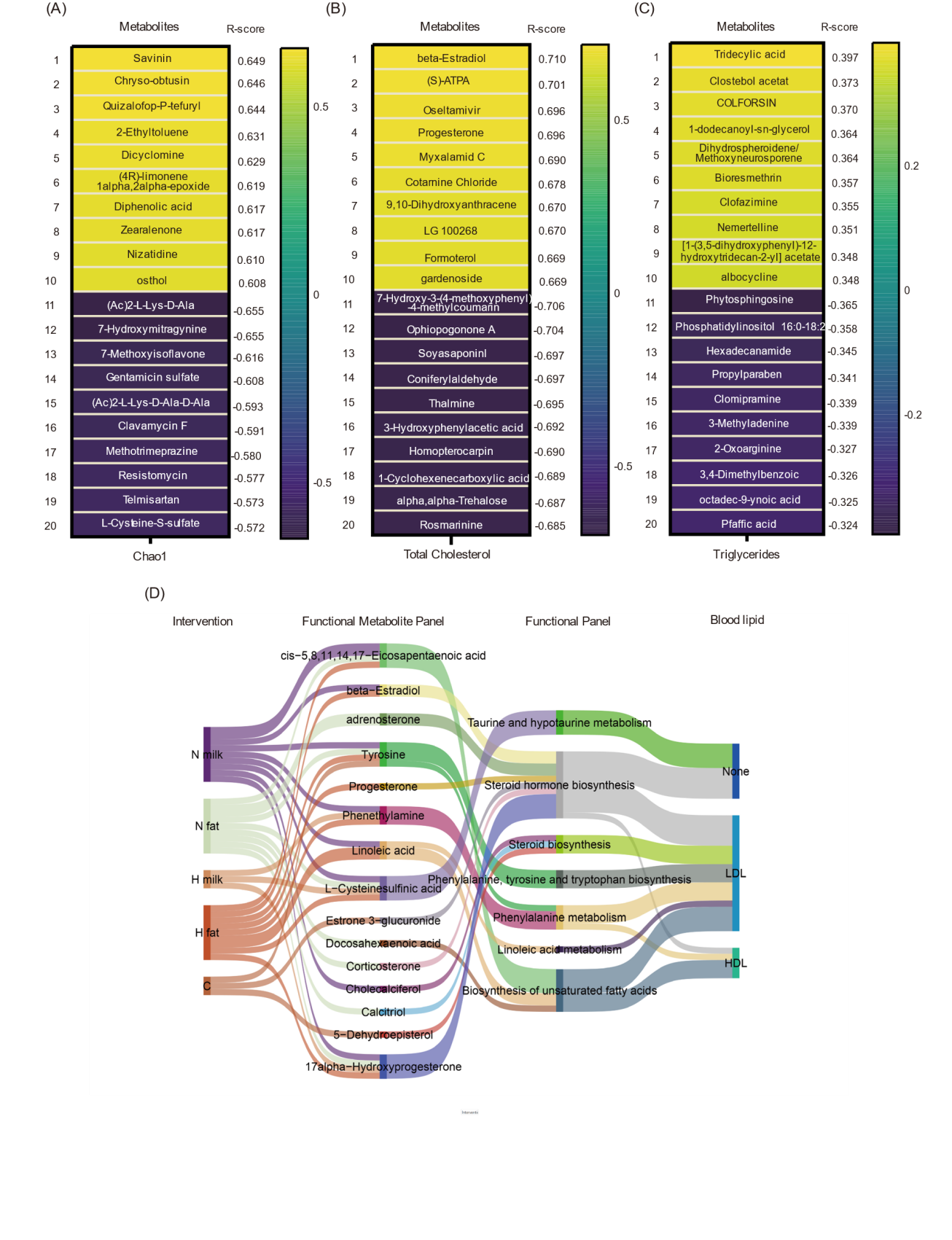


**Figure S3.** Effects of whole milk components on metabolites in ND and HFD mice and their relationship with lipid metabolism.

(A-C) The heatmap primarily displays the top 20 intestinal metabolites ranked by Spearman correlation coefficient R with host intestinal microbial diversity indices such as Chao1, total Cholesterol, and Triglycerides. (D) The Sankey diagram illustrates the differential functional metabolites caused by long-term intake of whole milk and dairy fat in the ND and HFD groups, as well as their corresponding regulatory relationships with HDL-C and LDL-C.

**
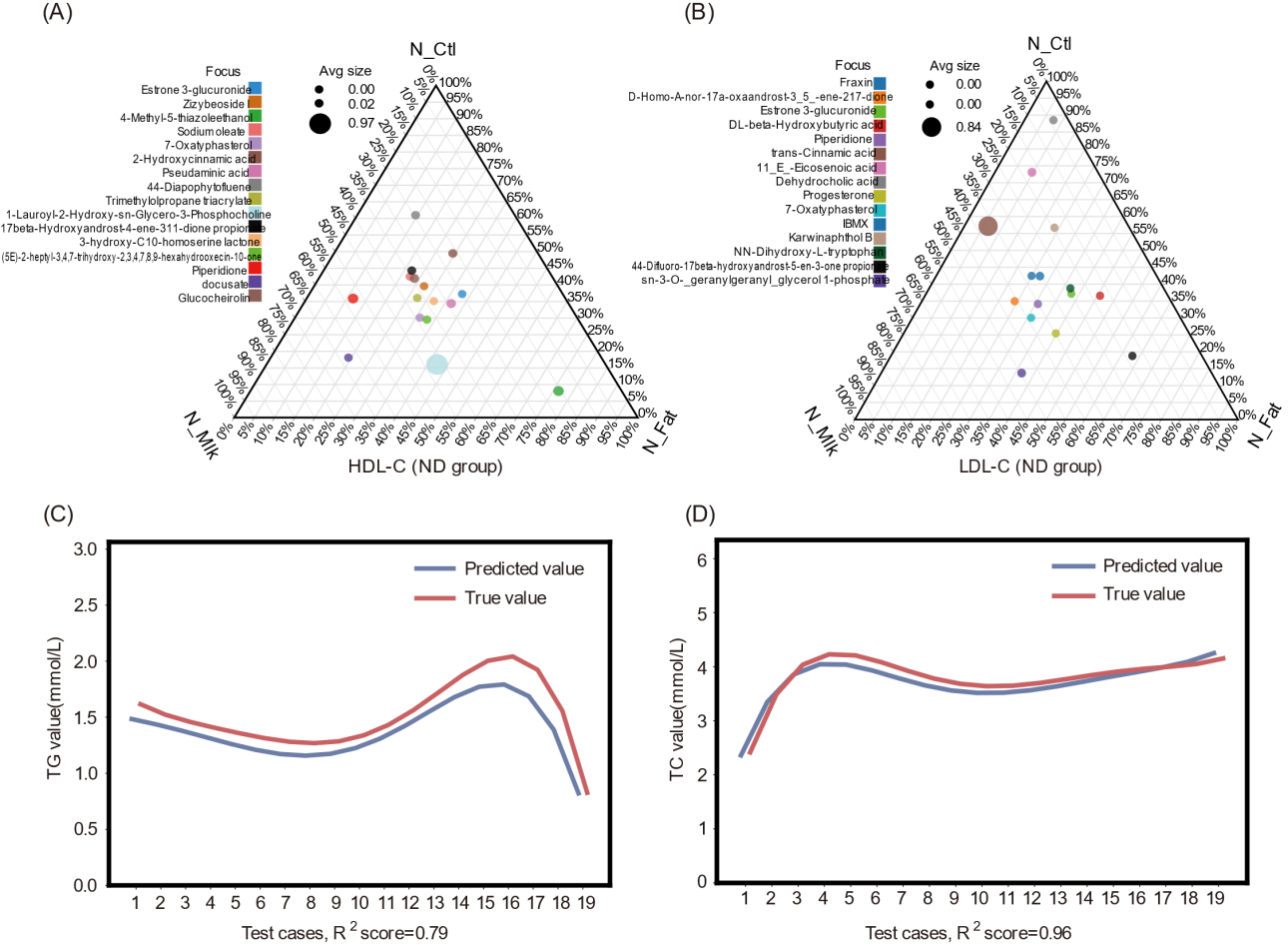
**

**Figure S4.** Prediction of blood lipids based on host gut metabolites. (A-B) The ternary plot primarily displays the concentrations of key metabolites for predicting HDL-C and LDL-C levels in the control group, whole milk group, and milk fat group. (C-D) Optimized machine learning algorithms can be accurately used for blood lipid prediction of triglycerides (TG) and total cholesterol (TC).


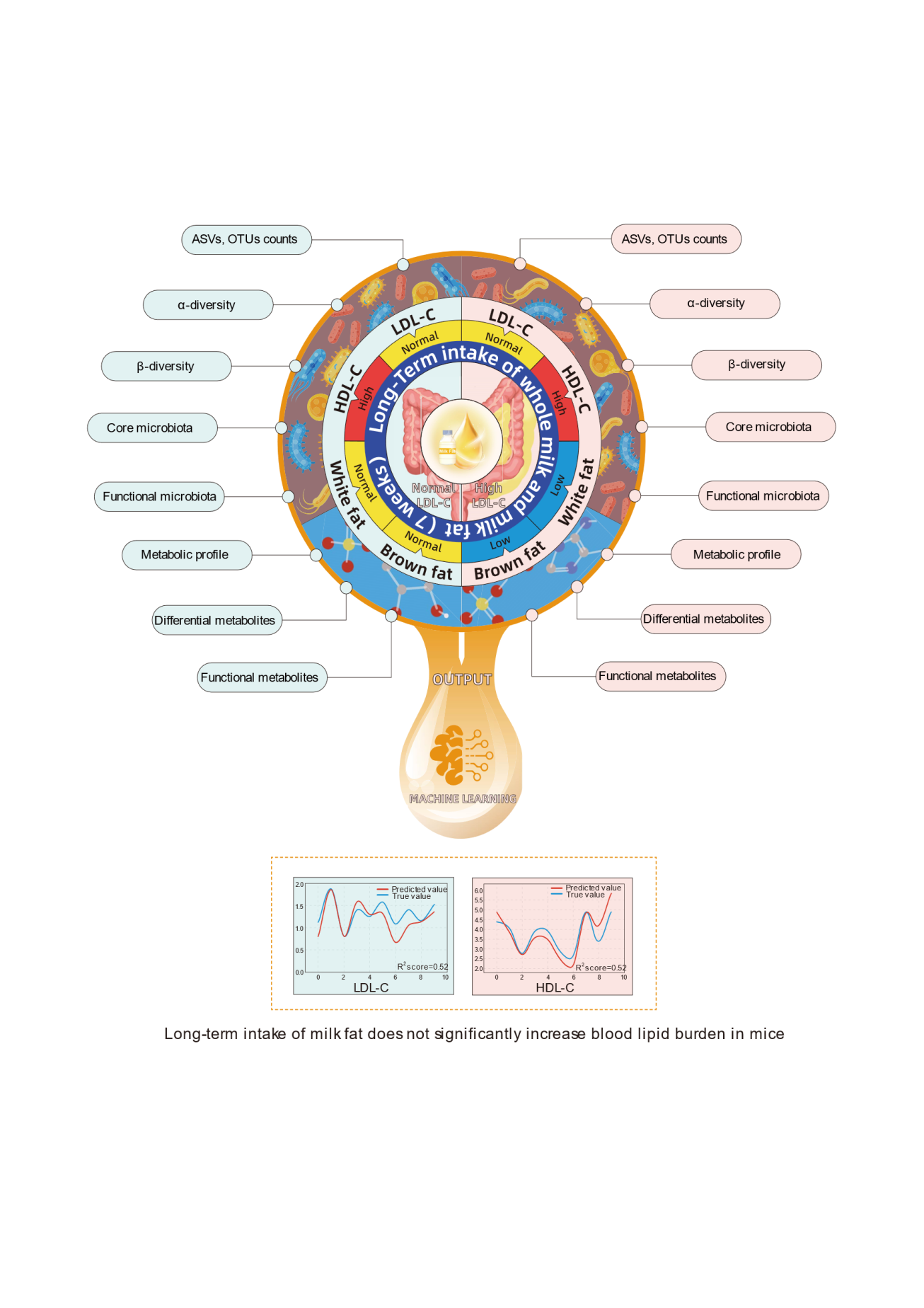
**Figure S5.** Overall summary diagram.

Normal and high-fat diet-fed mice did not experience increased blood lipid burden following a continuous 7-week intervention with milk fat and whole milk. Unlike dietary fats, milk fat can improve the diversity of the host’s gut microbiota and the composition of metabolites. The impact of a high-fat diet on the host’s gut microbes and metabolites is significantly greater than that of milk fat. Based on the host’s gut microbiota and metabolite composition, accurate predictions can be made regarding the changes in the host’s LDL-C and HDL-C levels.

## References

1. Ren, Guangxu, Jianping Zhang, Minghua Li, Zhenchuang Tang, Zhenni Yang, Guangyan Cheng, Jiaqi Wang. 2021. “Gut microbiota composition influences outcomes of skeletal muscle nutritional intervention via blended protein supplementation in posttransplant patients with hematological malignancies.” *Clinical Nutrition* 40: 94-102. <https://doi.org/https://doi.org/10.1016/j.clnu.2020.04.030>

2. Gao, Yunyun, Guoxing Zhang, Shunyao Jiang, Yong-Xin Liu. 2024. “Wekemo Bioincloud: a user-friendly platform for meta-omics data analyses.” *Imeta* 3: e175. <https://doi.org/https://doi.org/10.1002/imt2.175>
